# Comparison of hamstring and quadriceps tendon autografts in anterior cruciate ligament reconstruction with gait analysis and surface electromyography

**DOI:** 10.1186/s10195-021-00581-z

**Published:** 2021-05-21

**Authors:** J. Schagemann, T. Koebrich, R. Wendlandt, A. P. Schulz, J. Gille, R. Oheim

**Affiliations:** 1 Clinic for Orthopedics and Trauma Surgery, University Medical Center Schleswig Holstein UKSH Campus Luebeck, Ratzeburger Allee 160, 23538 Luebeck, Germany; 2 Clinic for Orthopedics and Trauma Surgery, Laboratory for Biomechanics, University Medical Center Schleswig Holstein UKSH Campus Luebeck, Ratzeburger Allee, 160, 23538 Luebeck, Germany; 3 BG Klinikum Hamburg, Bergedorfer Straße 10, 21033 Hamburg, Germany

**Keywords:** ACL reconstruction, Hamstring graft, Quadriceps tendon graft, Gait analysis, Surface electromyography

## Abstract

**Background:**

Anterior cruciate ligament (ACL) tear is the most frequent ligamentous injury of the knee joint. Autografts of hamstring (HS) or quadriceps tendons (QT) are used for primary ACL reconstruction. In this study, we planned to examine whether harvesting an HS graft is related to a deficit in dynamic knee stabilisation and strength revealed by dynamic valgus as compared with QT graft or the uninjured leg. Furthermore, if this deficit exists, is it compensated by higher neuromuscular activity of the quadriceps muscle?

**Materials and methods:**

Adult patients who had undergone ACL reconstruction with QT or HS autografts were included in this two-armed cohort study. Clinical outcome was assessed by clinical data analysis, physical examination and the Lysholm Score and Knee Injury and Osteoarthritis Score (KOOS). In addition, gait analysis and non-invasive surface electromyography were performed.

**Results:**

A complete data set of 25 patients (QT: *N* = 8, HS: *N* = 17) was analysed. There was no significant demographic difference between the groups. Time between surgery and follow-up was significantly longer for the QT group. Significant differences regarding clinical outcome were not found between the treated and untreated leg or between the two groups, with excellent scores at the time of follow-up. Gait analysis revealed no significant differences of varus–valgus angles. Significant differences in surface electromyography were only found in the QT group with increased vastus medialis obliquus activity of the treated legs (*p* < 0.01).

**Conclusions:**

Our results suggest that harvesting of HS grafts for primary ACL reconstruction will not lead to a medial collapse and consequently impaired medial stabilisation of the knee when compared with QT grafts.

**Level of evidence:**

IV.

## Introduction

Among those factors that impact clinical outcome after ACL reconstruction, graft selection might be the most critical yet controversial [1–3]. HS grafts are widely used and likely the current gold standard for primary ACL reconstruction [4–6]. The underlying rationale for this trend is a reliable and more anatomic fixation and a low rate of complications and resulting comorbidities [6, 7]. However, harvesting HS grafts eventually results in the impairment of flexor muscle strength and dynamic medial stabilisation [8–11]. This was demonstrated when HS grafts were compared with BPTB grafts [12] and QT grafts [13]. Since the ischiocrural muscles function as agonist of the ACL and as medial stabilisers, in theory, harvesting HS grafts could be a risk factor for recurrent injury. In contrast, patients who received HS grafts for primary ACL reconstruction had better extensor muscle strength compared with BPTB and QT collectives [13, 14]. Autologous HS and BPTB grafts have been thoroughly investigated, and both have been shown to provide comparable results regarding restoration of knee joint function and clinical outcome [15, 16]. Differences, however, seem to be slight, and both types of grafts are viable options for primary ACL reconstruction [15–20]. Another safe, versatile and suitable graft is the QT [21–23]. QT grafts in primary ACL reconstruction provide similar results to HS grafts without affecting comorbidities [24]. Lee et al. [10] showed that using either double-bundle HS or QT autografts resulted in similar functional outcome, yet better flexor muscle strength was observed in the QT group.

Although literature is comprehensive, the discussion about graft choice remains controversial [1, 2, 19, 21, 25]. Muscle strength might be a critical predictor of clinical outcome [26] and return to sports or preinjury activity levels. There is evidence that harvesting HS grafts weakens the agonists of the ACL and the MCL [9, 11], with the explanation that the hamstrings function as dynamic medial stabilisers [8]. Harvesting of HS grafts seems to result in strength deficits with knee flexion and inferior dynamic stability [12, 14, 27, 28]. In contrast, harvesting of QT or BPTB grafts weakens the quadriceps muscle.

Therefore, we planned to examine whether harvesting an HS graft is related to a deficit in dynamic knee stabilisation and strength revealed by dynamic valgus as compared with QT graft or the uninjured leg. Furthermore, the study was designed to reveal a potential strength deficit by compensatory neuromuscular activity of the quadriceps muscle.

## Materials and methods

### Inclusion criteria

Female and male patients, 18 years and older, who had received either a QT or HS autograph for ACL reconstruction in our department with a minimum follow-up period of 24 months were included in this cohort study. Additionally, to be included in this study, the patients were required to have the capability to walk on the treadmill for as long as 10 min. Poor compliance, previous surgeries on the knee, relevant comorbidities such as MCL injury or meniscal lesions at time of injury, and current injuries at time of follow-up were exclusion criteria. Concomitant injuries and pathologies representing exclusion criteria were monitored by physical examination and magnetic resonance imaging (MRI). To ensure consensus, participants had to complete a general anamnesis questionnaire.

### Ethical approval

All procedures involving human participants were in accordance with the ethical standards of the institutional and national research committee and with the 1964 Declaration of Helsinki and its later amendments or comparable ethical standards. The study was approved by the institutional ethical committee (AZ 14-296). Informed consent was obtained from all individuals, and participation was voluntary.

### Demographics

Overall, between 2008 and 2014, 202 patients were identified who had had ACL reconstruction in our department (QT: *N* = 51; HS: *N* = 152). In total, 103 patients met the inclusion criteria (QT: *N* = 23; HS: *N* = 80). Twenty-five patients participated in gait analysis (QT: *N* = 8; HS: *N* = 17), and were eligible for statistical analysis (QT: *N* = 8, female: *N* = 4, male: *N* = 4; HS: *N* = 17, female: *N* = 8, male *N* = 9). There were no significant differences between the QT and HS treatment groups regarding age (QT: 23 ± 12.96 SD years; HS 37 ± 11.66 SD years; *p* = 0.3565), height (QT: 175.6 ± 10.89 SD cm; HS: 177.3 ± 8.48 SD cm; *p* = 0.7093), weight (QT: 74.5 ± 16.97 SD kg; HS: 78.54 ± 15.05 SD kg; *p* = 0.5754) and body mass index (BMI; QT: 24.09 ± 4.17 SD kg/m^2^; HS: 24.97 ± 4.39 SD kg/m^2^; *p* = 0.6374).

### Clinical outcome

Clinical outcome assessment included measurement of the range of motion (ROM) and circumference of both the treated and contralateral intact leg. Leg circumference measurements were done according to the measuring sheet of the Deutsche Gesetzliche Unfallversicherung (DGUV) for lower extremities 20 cm and 10 cm above the joint line, at the middle of the patella, and 15 cm below the joint line. The smallest circumference of the lower leg was measured also. Prior to this, the dominant leg was identified. The anterior drawer test and the Lachman test were performed. The visual analogue scale (VAS) for pain level and the Lysholm and KOOS scores were used as patient-reported quantitative outcome measures. Graft failures and treatment associated complications were documented.

### Gait analysis

**Fig. 1:**
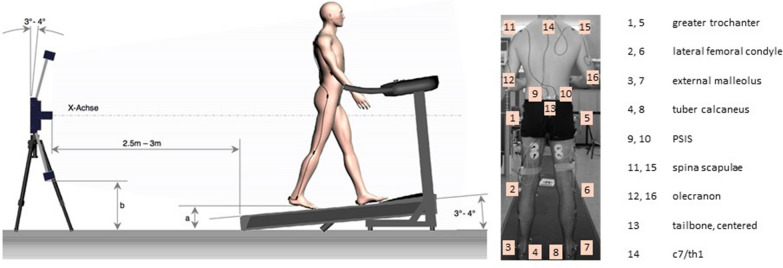
Schematic figure showing the layout of the gait analysis with the LUKOtronic Motion-Capture-Unit MCU200 (left); Picture with all the infrared marker positions shown from behind (right)

For the measurement of varus–valgus angles, the GaitLab system (Lutz Mechatronic Technology, Innsbruck, Austria) was used. The LUKOtronic Motion-Capture-Unit (MCU200) is equipped with infrared markers and sensors that allow for three-dimensional gait analysis. Infrared markers were fixed on the subjects as depicted in Fig. 1 (right). Subjects were guided through the system and were allowed to adapt to the *Calles* treadmill (Sprintex, Kleines Wiesental, Germany) for a couple of minutes (Fig. 1, left). Subsequently, measurements were started and conducted at both 4.5 and 6.0 km/h for as long as 5 min. Angles were measured between marker No. 1–3 (left leg) and No. 5–7 (right leg). The GaitLab software subdivides the individual gait cycle into 50 specific moments and enables calculation of varus (negative values) and valgus (positive values) angles. Initial contact (heel strike) was defined as the start of gait cycle, and terminal swing was defined as individual blank value. To enable inter-individual comparability, varus–valgus angles of the terminal swing phase were subtracted from the values of interest at loading response, terminal stance and mid-stance.

### Surface electromyography

Non-invasive surface electromyography was used to measure neuromuscular activity of the semitendinosus muscle, the VMO and the VLO. Electrodes (Version 2.11, Shimmer, Dublin, IR) were placed according to the recommendations of the *Surface ElectroMyoGrapy for the Non-Invasive Assessment of Muscles* (SENIAM) projects. Sensors and wires were fixed thoroughly to prevent motion artefacts. Prior to measurements, normalisation of maximum voluntary contraction (MVC) was performed to enable inter-individual and quantitative comparability [29]. Neuromuscular activity (µV) of VMO and VLO was measured using triple maximum isometric extension of the leg for 10 s (defined resistance). The semitendinosus muscle was tested the same way, but by triple maximum isometric flexion of the leg. In real time, data were transferred to a laptop via Bluetooth, using the Multi Shimmer Sync software to display the raw data and the DIAdem software (National Instruments, Munich, GER) to analyse the data.

### Statistical analysis

Statistical power analysis was based on a study by Tashman et al. [30]. The authors investigated *n* = 6 patients after ACL reconstruction using gait analysis for the measurement of valgus adduction [°] as major outcome measure. They found 2.8 ± 1.6° valgus adduction in ACL reconstructed limbs compared with healthy contralateral controls (0 ± 1.6°), resulting in an effect size of 1.75 (Cohens *d*). An a priori power analysis was conducted with G*Power (*t*-test: *α* = 0.05, power (1−β) = 0.85, effect size *d* = 1.75) [46]. According to our hypothesis and based on the referred study, we calculated a minimum of *n* = 7 per group. The data shown represent the means ± standard deviation (SD). The Mann–Whitney *U* test was run on clinical outcome data (Lysholm score and KOOS). Gait analysis and surface electromyography were analysed by the robust *t*-test of Yuen for differences and the two one-sided test (TOST) for equality using RStudio (Version 0.99.879; Boston, MA, USA). Gender differences were described by the *χ*^2^ test. Demographic data was compared by *t*-tests. The level of significance was set at *p* < 0.05.

## Results

### Clinical outcome

The mean follow-up of the QT group was 68.6 months, and of the HS group 31.1 months. At individual follow-ups, there were no significant differences in range-of-motion measurements between the treated and the contralateral intact legs and between the two treatment groups. The same was true for leg circumference measurements (*p* = 0.9673–0.1039). Pain based on VAS was 0.75 ± 1.39 SD in the QT group and 0.35 ± 0.86 SD in the HS group. The difference between the two groups was not statistically significant (*p* = 0.4746). Functional scores for both groups were excellent at time of follow-up (QT: Lysholm: 86.88 ± 10.09 SD, KOOS: 91.32 ± 8.44 SD; HS: Lysholm: 92.06 ± 9.32 SD, KOOS: 91.04 ± 8.22 SD). The overall difference between functional scores and differences between subcategories of select scores were not statistically significant (overall Lysholm score *p* = 0.18; overall KOOS score *p* = 0.821), except for a slight but significant advantage for the HS group for the Lysholm score item ‘climbing stairs’ (QT: 9.0 ± 1.85 SD; HS: 10 ± 0.0 SD; *p* = 0.04).

### Gait analysis

**Fig. 2:**
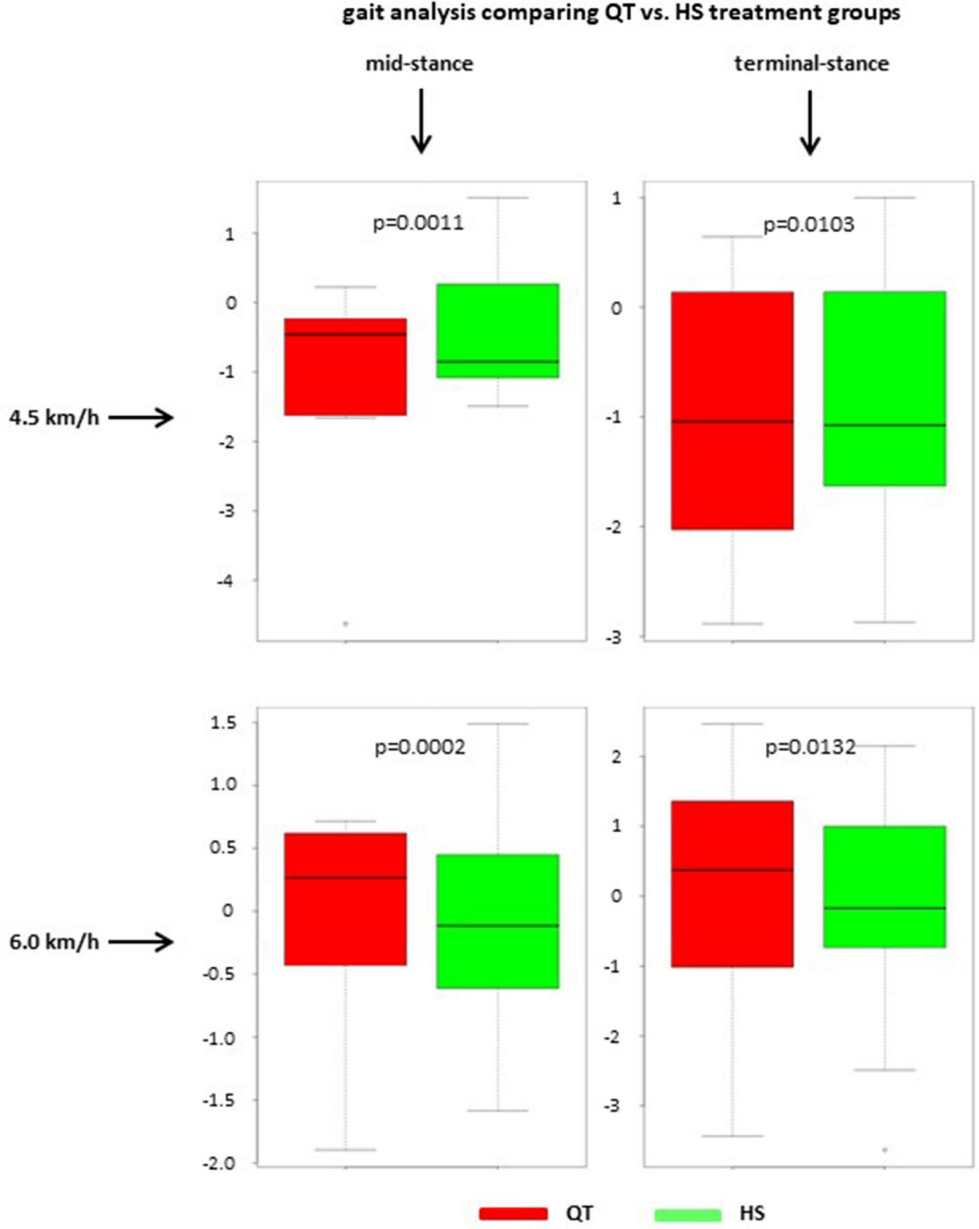
Boxplot charts comparing the varus–valgus angles between the QT (red) and HS (green) group during mid- and terminal stance at 4.5 km/h and 6.0 km/h. All four boxplot charts show significant equality between the treated knees of both groups

Gait analysis revealed no significant differences of varus–valgus angles when comparing treated and contralateral intact legs. This was independent of the choice of transplant (QT or HS). These findings applied for loading response, mid-stance and terminal stance both at 4.5 km/h and 6.0 km/h. For detailed yield and *p*-values, see Table 1. When comparing the two different treatment groups (QT versus HS), there were no significant differences in varus–valgus angles measured at loading response, mid-stance and terminal stance both at 4.5 km/h and 6.0 km/h. For detailed yield and *p*-values, see Table 2. This was further evaluated, and a significant equality between the treated QT and HS group in varus–valgus angles measured at mid-stance and terminal stance both at 4.5 km/h (*p* = 0.0011; *p* = 0.0103) and 6.0 km/h (*p* = 0.0002; *p* = 0.0132) could be proven (Fig. 2).

**Table 1:** Mean values and standard deviations of the varus–valgus angles for the treated and contralateral intact knees in the QT and HS group during the loading response, mid-stance and terminal stance at 4.5 km/h and 6.0 km/h

		Treated	Control	*N*	*p*
QT					
4.5 km/h	Loading response	−1.48 ± 2.09	0.81 ± 0.99	8	0.65
	Mid-stance	−1.13 ± 1.56	−0.84 ± 1.38	8	0.9
	Terminal response	−1.02 ± 1.28	−1.06 ± 1.66	8	0.93
6.0 km/h	Loading response	−0.29 ± 0.77	−0.49 ± 0.67	8	0.63
	Mid-stance	−0.03 ± 0.89	−0.1 ± 0.18	8	0.99
	Terminal response	−0.06 ± 1.87	−0.18 ± 1.54	8	0.95
HS					
4.5 km/h	Loading response	−0.16 ± 0.92	−0.15 ± 0.94	17	0.2
	Mid-stance	−0.36 ± 0.93	−0.1 ± 1.08	17	0.17
	Terminal response	−0.83 ± 1.27	−0.54 ± 1.11	17	0.5
6.0 km/h	Loading response	−0.18 ± 0.92	0.14 ± 0.87	16	0.28
	Mid-stance	−0.03 ± 0.9	0.36 ± 0.95	16	0.09
	Terminal response	−0.1 ± 1.54	0.55 ± 1.17	16	0.23

**Table 2:** Mean values and standard deviations of the varus–valgus angles for the treated knee in the QT and HS group during the loading response, mid-stance and terminal stance at 4.5 km/h and 6.0 km/h

		QT		HS		
		Treated	*N*	Treated	*N*	*p*
4.5 km/h	Loading response	−1.48 ± 2.09	8	−0.16 ± 0.92	17	0.2388
	Mid-stance	−1.13 ± 1.56	8	−0.36 ± 0.93	17	0.5058
	Terminal response	−1.02 ± 1.28	8	−0.83 ± 1.27	17	0.7319
6.0 km/h	Loading response	−0.29 ± 0.77	8	−0.18 ± 0.92	16	0.8109
	Mid-stance	−0.03 ± 0.89	8	−0.03 ± 0.9	16	0.4902
	Terminal response	−0.06 ± 1.87	8	−0.1 ± 1.54	16	0.7572

### Surface electromyography

**Fig. 3:**
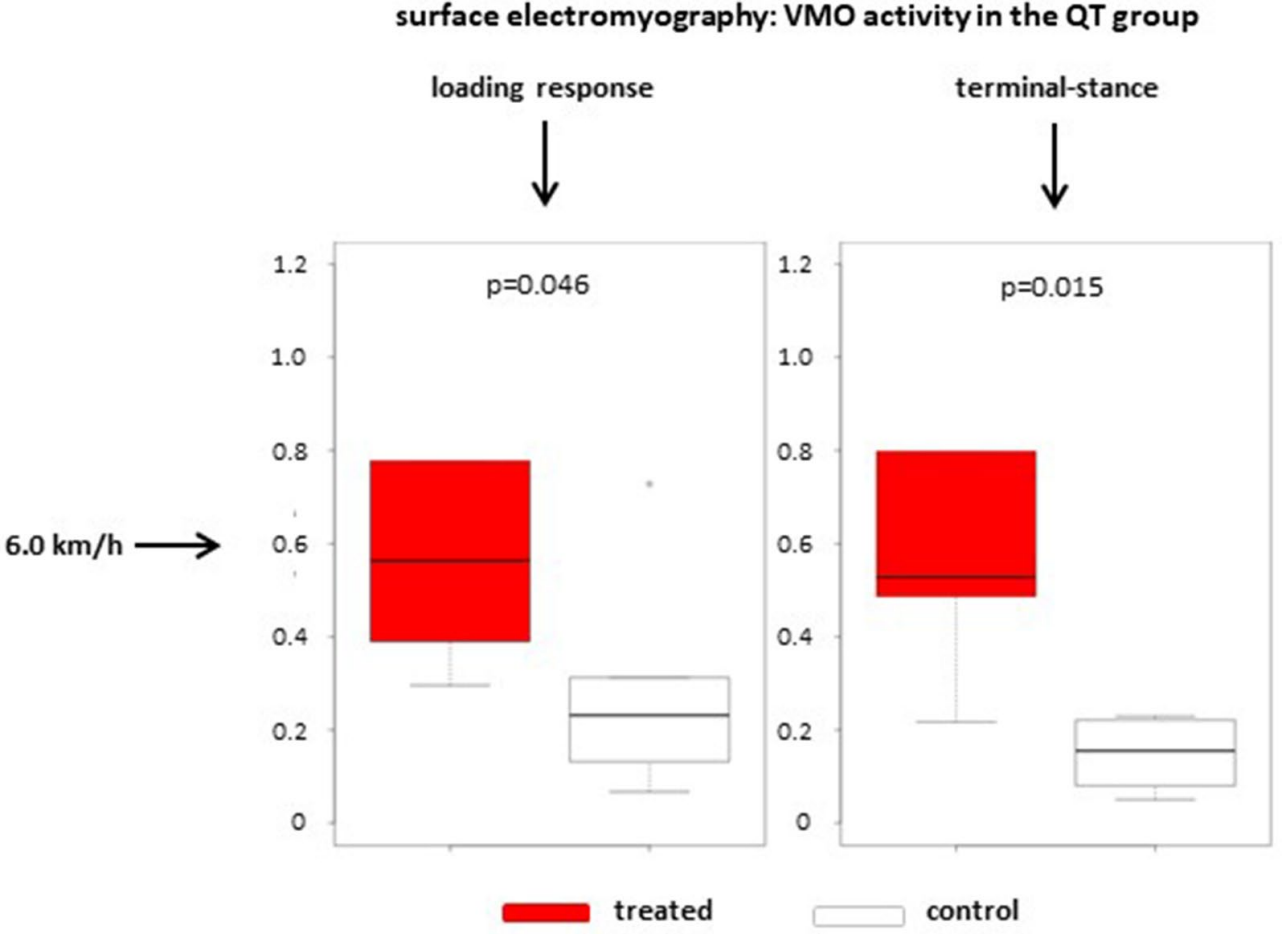
Boxplot charts showing VMO activity (µV) for the treated and contralateral side in the QT group during loading response and terminal stance at 6.0 km/h. In both phases, there is a significant difference between the treated and contralateral intact side

**Fig. 4:**
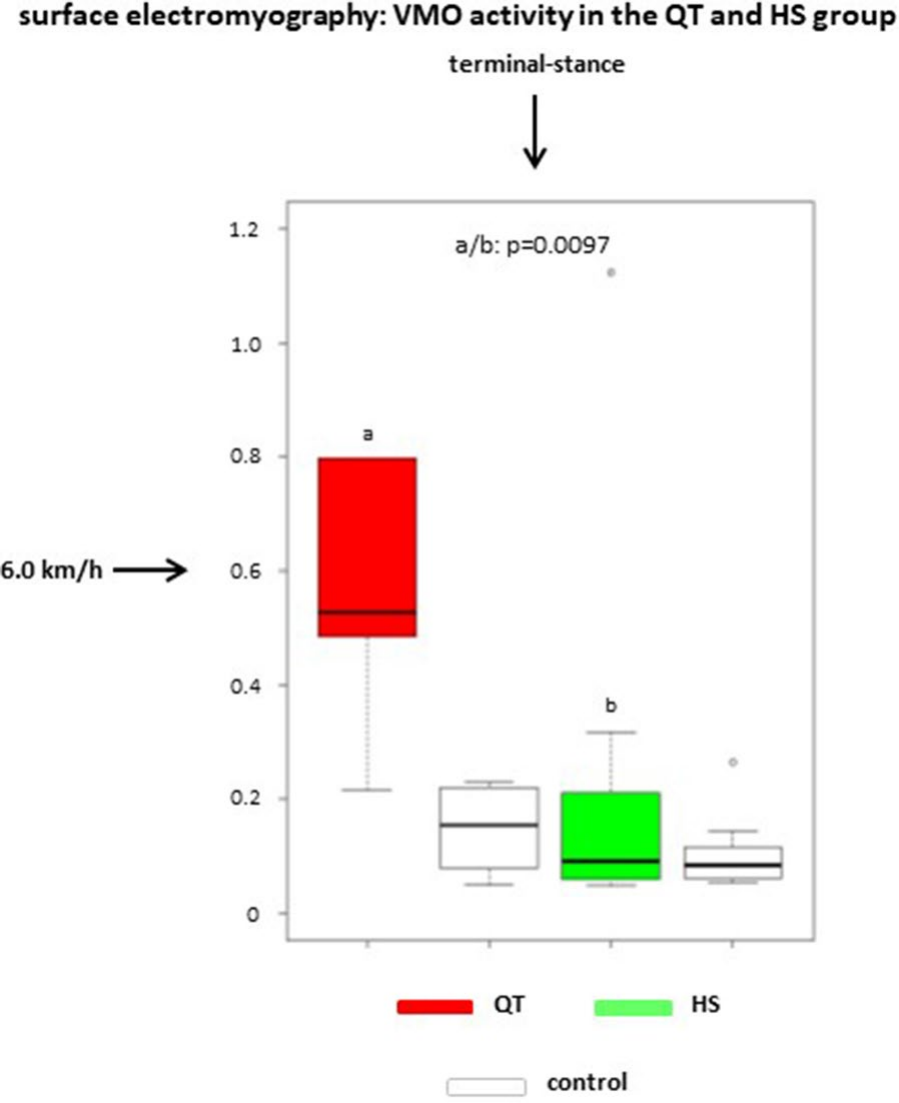
Boxplot chart showing VMO activity (µV) for the treated and contralateral side in the QT and HS groups during terminal stance at 6.0 km/h. There is a significant difference between the QT and HS groups regarding the treated legs

Surface electromyography was used to measure neuromuscular activity of the semitendinosus muscle, the VMO and the VLO. At 4.5 km/h, five QT cases and six HS cases were analysed, and six QT cases and seven HS cases were analysed at 6.0 km/h. In the QT group, there was one significant difference between the treated and contralateral intact legs with respect to VMO activity measured at loading response and terminal stance at 6.0 km/h (loading response: *p* = 0.046; terminal-stance: *p* = 0.015, Fig. 3). There were no other significant differences in the QT group. In the HS group, surface electromyography did not reveal any significant differences when comparing treated and contralateral intact legs, three different muscles and different stance phases. When comparing the treated legs of the QT and the HS groups, we found a significant difference (*p* = 0.0097) in VMO activity at terminal stance at 6.0 km/h (Fig. 4) with increased activity in the QT group and normal activity comparing in the HS group comparing the treated and contralateral intact legs. Other differences were not statistically significant.

## Discussion

Biomechanical studies implementing gait analysis have primarily focused on sagittal knee joint kinematics of ACL deficient and reconstructed knees [31, 32]. Data on dynamic changes in frontal knee joint angles comparing two different grafts have been analysed less often [31–33]. Reconsideration of dynamic stability after ACL reconstruction is not trivial because a dynamic valgus is a crucial predictor for clinical outcome and the return to sports or preinjury activity [26]. According to Abrams et al., harvesting HS grafts may result in strength deficits with knee flexion compared with BPTB-treated collectives [12]. Mohammadi et al. showed that HS grafts for ACL reconstruction performed better in terms of quadriceps strength in comparison with patients in the BPTB group [14]. Patients after ACL reconstruction had reduced quadriceps and hamstring strength and inferior dynamic stability, including hop performance and jump-landing strategy [14, 27, 28]. There is evidence that harvesting HS grafts weakens the agonists of the ACL and the main medial stabilizer MCL [9, 11], with the explanation that the hamstrings function as dynamic medial stabilisers [8]. Dynamic valgus indicates impaired medial stabilisation and consequently higher risk for re-injury. Comparing two different surgical techniques helps in deciding whether the current gold standard, HS graft [4], particularly in athletes [5], or other well-known grafts such as QT or BPTB grafts should be selected [2, 3].

In our study, both ACL reconstruction techniques (QT and HS) provided excellent clinical outcome, which is in line with other studies [34–37]. Gait analysis also proved both ACL reconstruction techniques to be successful. We could not find any significant difference between the QT and the HS groups. Moreover, we could not find any difference in frontal knee joint kinematics during walking compared with healthy contralateral controls. This is worth mentioning because prevailing dynamic valgus might have indicated muscular deficits due to the choice of implant. As for our hypothesis, this was true neither for the QT nor the HS group.

We could not confirm that the anticipated HS graft-related strength deficit would be compensated by higher neuromuscular activity of the quadriceps muscle. The opposite was true as treated legs in the QT group revealed a significantly higher VMO activity compared with healthy contralateral legs in the QT group and treated legs in the HS group. In contrast, surface electromyography in the HS group did not reveal any significant differences when comparing treated and contralateral intact legs. A higher neuromuscular activity of the VMO at terminal stance was found in the QT group, whereas Perry et al. described that there is usually no VMO activity at terminal stance in healthy collectives [38].

Xergia et al. summarized that isokinetic muscle strength deficits after ACL reconstruction are linked to the location of the donor site [39]. Ageberg et al. suggested that the strength deficit of the hamstrings and the lower hamstring-to-quadriceps ratio after HS harvesting will impair dynamic knee joint stabilisation [8]. Other studies showed that there is a strength deficit after harvesting BPTB or HS grafts even 5 years post ACL reconstruction [26, 40]. This evidence conflicts with our results, as enhanced neuromuscular activity of thigh muscles or dynamic valgus was not observed in the HS group, indicating either compensation of strength deficit or lack of medial stabilisation. A mild varus kinematic rather than dynamic valgus in both ACL reconstruction groups was found. Although mild varus kinematic cannot generally be equated to varus thrust, it is suspected to be a major reason for ACL reconstruction failure [41] and should be subject to further investigation, with respect to collective specific re-rupture rates.

We found increased VMO activity in the QT group, in line with a publication by Iriuchishima et al. [42], which showed quadriceps hypotrophy within 6 months after surgery, although hypotrophy had recovered after 12 months. This is remarkable because the mean follow-up of our QT group was 68.6 months compared with 31.1 months in the HS group. Thus, we could have expected a recovery especially of the QT group as described by Iriuchishima et al. [42]. Higher neuromuscular activity can also be interpreted as a compensatory mechanism for the strength deficit yet is not equal to muscle strength. In contrast, Bryant et al. concluded that subjects after ACL reconstruction demonstrated enhanced motor unit recruitment reflective of reduced quadriceps muscle fibre atrophy. This is coupled with increased quadriceps strength and musculotendinous stiffness of the lower limb musculature [43].

In conclusion, our results suggest that harvesting HS grafts for primary ACL reconstruction may not affect dynamic medial stabilisation. Our results are supportive of the use both of QS and of HS grafts for primary ACL reconstruction [36, 44]. However, graft fixation, gender, additional injuries and donor site morbidity have to be considered when deciding which graft to choose [20, 45]. Shortcomings of the present study are the small population, which is primarily due to the lifelike heterogeneity and concomitant injuries particularly of the MCL, which are prevalent in approximately one-third of all ACL cases. These cases had to be excluded. Moreover, dynamic valgus is multifactorial, and hip abductor, knee rotation and foot pronation issues should also be considered. Cadaveric cutting studies may have the potential to answer this eventually.

## Data Availability

The data sets used and/or analysed during the current study are available from the corresponding author on reasonable request. Besides, all data generated or analysed during this study are included in this published article.

## Abbreviations

ACL: Anterior cruciate ligament
QT: Quadriceps tendon
HS: Hamstrings
VMO: Vastus medialis obliquus
BPTB: Bone patella tendon bone
AZ: Aktenzeichen
SD: Standard deviation
BMI: Body mass index
ROM: Range of motion
KOOS: Knee injury and osteoarthritis outcome score
VAS: Visual analogue scale
VLO: Vastus lateralis obliquus
